# Immunity to glycan α-Gal and possibilities for the control of COVID-19

**DOI:** 10.2217/imt-2020-0247

**Published:** 2020-12-14

**Authors:** José de la Fuente, Christian Gortázar, Alejandro Cabezas-Cruz

**Affiliations:** ^1^SaBio. Instituto de Investigación en Recursos Cinegéticos IREC-CSIC-UCLM-JCCM, Ronda de Toledo s/n, 13005 Ciudad Real, Spain; ^2^Department of Veterinary Pathobiology, Center for Veterinary Health Sciences, Oklahoma State University, Stillwater, OK 74078, USA; ^3^UMR BIPAR, INRAE, ANSES, Ecole Nationale Vétérinaire d'Alfort, Université Paris-Est, Maisons-Alfort 94700, France

**Keywords:** α-gal, covid-19, glycan, immunity, microbiota, pandemic, probiotic, SARS-CoV-2, tick, vaccine

Ongoing research focuses on vaccines targeting SARS-CoV-2 for the prevention and control of COVID-19. However, boosting the immune response to glycan α-Gal with a broader and not restricted to pathogen-specific immunity may contribute not only to the control of COVID-19 but also to other pathogens that negatively affect the individual response to SARS-CoV-2.

The COVID-19 pandemic has expanded rapidly worldwide with millions of infected individuals and hundreds of thousands dead patients. Recently, new vaccines are under investigation at different levels for the control of COVID-19 [1]. These vaccines are focused on the SARS-CoV-2 by using inactivated or attenuated whole viruses, replicating and nonreplicating vectors, virus-like particles, recombinant proteins and RNA/DNA technologies [1]. However, despite the advances and potential impact that these vaccines represent for the prevention and control of COVID-19, interventions boosting the immune response with a broader and not pathogen-specific immunity may contribute not only to the control of COVID-19 but also to potential reinfections by SARS-CoV-2-related genetic variants or other pathogens that negatively affect the individual response to SARS-CoV-2.

## The α-Gal syndrome (AGS)

The AGS is a disease associated with tick salivary glycoproteins with Galα1-3Galβ1-(3)4GlcNAc-R (α-Gal) modifications that in some individuals exposed to tick bites results in the production of anti-α-Gal IgE antibodies, which may cause delayed anaphylaxis to mammalian meat consumption or immediate anaphylaxis to tick bites, xenotransplantation and certain drugs such as cetuximab [2]. As an evolutionary adaptation, humans lost the capacity to synthesize the glycan α-Gal to have the capacity of boosting the protective immune response against pathogens containing this glycan on their surface with the trade-off of a higher risk to AGS [2,3]. It was proposed that due to viral epidemics caused by viruses containing α-Gal, a catastrophic-selection event resulted in Old-World monkeys and apes lacking α-Gal epitopes and thus producing natural antibodies against this glycan to control pathogen infection [3]. Pathogens containing α-Gal on their surface include but are not limited to *Plasmodium*, *Leishmania*, *Mycobacterium*, *Aspergillus*, *Trypanosoma*, C retrovirus, porcine endogenous retrovirus, lymphocytic choriomeningitis virus, newcastle disease virus, sindbis virus, vesicular stomatitis virus, HIV, measles virus, paramyxovirus or vaccinia virus [2,3]. The proposed immune mechanisms associated with the AGS include TLR-mediated responses in both Th1 and Th2 cells and with a possible role for basophils in this process [4].

## The immune response to α-Gal

In humans, natural anti-α-Gal antibodies are produced in response to bacteria present in the gut microbiota and containing this modification on their surface [5]. The anti-α-Gal IgM/IgG antibodies are abundant (e.g., up to 1% of all circulating IgGs may be against α-Gal [6] and can be protective by opsonizing pathogens with α-Gal on their surface [2,5]. However, in response to α-Gal additional immune-mediated mechanisms such as macrophage response, complement system, B-cell maturation and upregulation of pro-inflammatory cytokines through the NF-κB innate immune pathway may be also activated [7,8]. The TLR signaling induces the Nrf2 signaling pathway, which is an important component in host anti-inflammation defence [9].

## How the immune response to α-Gal may contribute to the control of COVID-19

Co-infections with SARS-CoV-2 and other pathogens and the re-infection with SARS-CoV-2 could affect individual immune response and cause health conditions that may increase the risk of disease. Therefore, by increasing the immune response to α-Gal through the activation of different immune mechanisms it would be possible to a have a multivariable outcome acting at different stages of COVID-19 (Supplementary Figure 1). First, it would be possible to limit the zoonotic transmission of SARS-CoV-2 by complement-mediated and antibody-mediated opsonization if the virus contains α-Gal on its surface [3]. The reservoir and intermediate SARS-CoV-2 host animal species have not been identified, but existing evidence suggest that bats may be reservoir hosts and carnivores such as pangolins and cats may be involved in virus zoonotic transmission [10]. Human-to-human transmission could also be limited by anti-α-Gal antibodies. SARS-CoV-2 replication in blood type B individuals could generate viral particles containing the antigen B in their glycosylated envelops [11]. Antigen B is structurally related to α-Gal and crossreacts with anti-α-Gal Abs [12]. As expected, antibodies that crossreact with antigen B were observed only in B-negative individuals [12]. Thus, individuals of blood type A or O with high levels of anti-α-Gal and anti-B antibodies could be protected from SARS-CoV-2 particles generated from blood type B/AB individuals. Furthermore, anti-α-Gal antibodies crossreact with other glycans such as α-Galactosyl-mimotopes and thus may interfere with infection by pathogens without α-Gal in their capsule polysaccharides [13]. Then, and independently of the virus α-Gal content, the control of COVID-19 may be obtained through boosting protective immune responses mediated by α-Gal together with a higher protection to pathogens that negatively affect the individual response to SARS-CoV-2 and with α-Gal or crossreactive epitopes on their surface [8].

It has been shown that the infection with SARS-CoV-2 potentially can result in acute respiratory distress syndrome and other pathologies due to the ‘cytokine storm syndrome (CSS)’ [14]. The CSS has been associated with the activation of the NF-κB innate immune pathway resulting in the upregulation of pro-inflammatory cytokines such as TNF-α, IL-1β, IL-6 and IL-8 [14]. Consequently, treatment with N-acetylcysteine, alpha lipoic acid and Nrf2 activators may be used for blocking NF-κB as a novel treatment for the acute respiratory distress syndrome and CSS in patients with COVID-19 [12]. In this context, the immunization with α-Gal has resulted in the overexpression of IL-1β but not TNF-α, together with the upregulation of heat shock proteins, ribosomal proteins and the TLR2 pathway that activates Nrf2-mediated anti-inflammatory responses [8]. How α-Gal mediates the balance between immune pro-inflammatory and anti-inflammatory responses has not been established but based on immunization trials with α-Gal in animal models (e.g., [8]), the hypothesis is that the activated immune system balance pro- and anti-inflammatory cells and signals [15]. Taken together, these results suggest that the immunization with α-Gal may also contribute to decrease the inflammatory response and the levels of some pro-inflammatory cytokines such as TNF-α to address the CCS in COVID-19 patients. Glycans play a role in innate immunity and results in zebrafish treated with tick saliva showed the activation of innate immune responses mediated by upregulation of complement C3 in intestine macrophages [4]. If reproduced in humans, then α-Gal alone or in combination with other biomolecules could induce trained immunity trough C3 and other mechanisms, which may protect against pathogen infection.

Nevertheless, the natural antibody response to α-Gal is not only sufficient for host immunity but may be affected by SARS-CoV-2 infection. Recently, we characterized the antibody response to α-Gal in patients with COVID-19 at different disease stages in comparison with healthy controls [16]. The results showed that the increase in anti-SARS-CoV-2 IgG antibody titers and inflammatory responses correlated with the reduction in anti-α-Gal antibody titers (IgE, IgM, IgG) and the alteration of anti-α-Gal antibody isotype composition. These results suggested that the inhibition of the α-Gal-induced immune response correlates with COVID-19 severity by translating into more aggressive viremia and severe disease inflammatory symptoms [16].

The immunization with α-Gal could be done through the oral/mucosal route by the use of probiotics or postbiotics with high α-Gal content [17]. In addition to the immune response elicited by α-Gal immunization, the probiotics may also improve oral and lung microbiotas and exert bacterial interference against pathogens and promote IFN-γ production. Recent results have shown that in COVID-19 patients, the microbiota suffers dysbacteriosis with enrichment of opportunistic pathogens and depletion of beneficial commensals [18]. These preliminary results support the development of interventions to modify the microbiota and reduce the severity of COVID-19. For example, probiotics based on α-Gal-positive bacteria such as *Streptococcus* spp. that are predominant commensal species in the eubiotic microbiota (e.g., *Streptococcus salivarius*) may constitute good interventions against COVID-19 and other infectious diseases [19]. Finally, probiotic/postbiotc-based formulations have a low production cost and are easy to administer in regions with limited access to health services could have a major impact on the prevention and control of COVID-19 and other major infectious diseases worldwide.

## Conclusion

Despite the advances and potential impact that new vaccines represent for the prevention and control of COVID-19, interventions boosting the immune response to α-Gal with a broader and not pathogen-specific immunity may contribute not only to the control of COVID-19 but also to potential re-infections by SARS-CoV-2-related genetic variants or other pathogens that negatively affect the individual response to SARS-CoV-2. Based on the results regarding the immune response to α-Gal we propose the development of probiotic/postbiotc-based formulations with abundant commensal bacteria of the gut and lung microbiota with high α-Gal content for the prevention and control of COVID-19 and other major infectious diseases worldwide [20,21]. Furthermore, vaccines for the control of COVID-19 through induction of anti-α-Gal protective responses may be obtained by producing coronavirus or virus-like particles in noncatarrhine mammalian cells [22,23]. One of the major challenges of vaccination campaigns in poor regions with high prevalence of infectious diseases is the distribution and administration of the vaccine. The possibility of developing probiotic/postbiotic that can be delivered in stable formulations such as a yogurt or food supplements, will make these interventions easier to distribute and administer. These formulations have a low production cost and are easy to administer with a major impact in regions with limited access to health services.

To address the limitations of these hypotheses, future research should explore the α-Gal/antigen B/anti-α-Gal antibodies crossreactive epitopes content in SARS-CoV-2 and other coronaviruses, the spread of COVID-19 and other coronavirus-caused diseases via intermediate animal hosts, the mechanisms mediated by α-Gal immunization to balance immune pro-inflammatory and anti-inflammatory responses, the characterization of gut and lung microbiota including α-Gal content in patients at different stages of COVID-19, the identification of commensal bacteria with α-Gal modifications for the development and evaluation of probiotic/postbiotc-based formulations and the impact of other host and virus derived factors that may affect the severity of COVID-19 [24].
